# “Not doing it justice”: Perspectives of Recent Family Medicine Graduates on Mental Health and Addictions Training in Residency

**DOI:** 10.1177/23821205241238642

**Published:** 2024-04-09

**Authors:** Abigail Ramdawar, Nikki Bozinoff, Kimberly Lazare

**Affiliations:** 1 206712 Institute of Health Policy Management and Evaluation, University of Toronto, Toronto, ON, Canada; 2 204352 Department of Family and Community Medicine, University of Toronto, Toronto, ON, Canada; 3 Campbell Family Mental Health Research Institute, Centre for Addiction and Mental Health, Toronto, ON, Canada; 4 8613 North York General Hospital, Toronto, ON, Canada

**Keywords:** curriculum, mental health, residency, family medicine, professional identity

## Abstract

**OBJECTIVES:**

Family physicians report feeling inadequately prepared to meet the evolving mental health care needs of the population. Little scholarship exists evaluating the effectiveness of curricula designed to teach mental health and addiction (MH&A) care to family medicine (FM) residents. As such, the purpose of this study was to explore the experiences of recent FM residency graduates in providing mental health care, and their perceptions of mental health training gaps during their residencies.

**METHODS:**

A qualitative descriptive study design was conducted by 8 recent graduates of the University of Toronto's FM residency program, who participated in semi-structured video interviews. A thematic analysis approach was used to collect and analyze the data.

**RESULTS:**

Through thematic analysis, 3 overarching themes were developed: (1) barriers in providing mental health and addiction care, (2) curriculum renewal, and (3) the role of FPs and professional identity. Consistent with the literature, the majority of recent FM graduates expressed discomfort when managing patients with mental health and addiction concerns. Additionally, participants perceived residency program time constraints, rotational site differences, and limited exposure to marginalized populations all impacted learning and mastery of skills.

**CONCLUSION:**

The findings of this study underscore current gaps within the FM residency curriculum and highlight the need to address current curricular deficits.

## Introduction

Mental illness is a leading cause of disability in Canada with 1 in 5 Canadians experiencing mental health concerns.^1–3^ In Canada, a variety of clinicians see patients with mental health concerns, including family physicians (FPs), psychiatrists, psychologists, psychotherapists, nurses, social workers, and counselors.^4^ FPs, however, are often patients’ first point of contact with the healthcare system and provide almost two-thirds of mental health services.^5–7^ In 2022, 48.8% of patients who met the criteria for a mood, anxiety, or substance use disorder reported that they had talked to a health professional about their mental health in the past year, with 32.4% reported speaking with an FP, 12.7% to a psychiatrist, and 21.0% to a psychotherapist, social worker, or counselor).^8^ It is estimated that 50% of FPs’ time is devoted to mental health problems.^8^ Despite this, many FPs feel inadequately prepared to meet the evolving mental health needs of the population.^1,7,9^ If an FP feels that their patient requires more specialized care, they may refer their patient to Psychiatry or Addiction Medicine.^4^ Most of the counseling and/or psychotherapy services in Canada are provided privately with patients left to shoulder out-of-pocket costs, which can be a barrier for many.^8^ With long wait lists to access specialized mental health care, many FPs feel isolated in managing these complex medical conditions.^10,11^ A recent retrospective cohort study of a large community-based sample of patients in Toronto, Canada, reported the median wait time from the date of referral to the date of psychiatric consultation was 133 days.^12^

Although FPs provide the majority of mental health and addiction care, studies suggest that when their care is compared to evidence-based guidelines, the quality of care provided by FPs to patients with mental health concerns is suboptimal, there is inadequate follow-up, and no significant improvement in long-term health outcomes.^13,14^ Underpinning feelings of unpreparedness to manage common mental health disorders like anxiety and depression, many FPs acknowledge current gaps and deficits in their training and knowledge of mental health topics.^15,16^ For example, a 2007 survey of 163 FPs in Ontario revealed that 80% had received no training in cognitive behavioral therapy (CBT) or knew little about it.^5^ Furthermore, in Canada, psychotherapy training for family medicine (FM) residents is presently absent in the formal curriculum.^17^ The degree of preparedness and comfort in providing patient care in a specific area is largely influenced by the FPs’ levels of self-efficacy and degree of educational exposure (ie, residency training).^18^ In their *Outcomes of Training project*, the College of Family Physicians of Canada (CFPC) identified mental health and addiction training as both an area of emerging importance and one that requires educational enhancement and priority attention.^18^

Furthermore, very little scholarship exists on curricula designed to teach mental health care to FM residents,^19^ with mental health training in FM residency programs varying significantly in Canada, both in quantity and quality.^20^ Although many Canadian FM residency programs have dedicated curricula in this area, few studies have explored the effectiveness of these curricula. In the United States (US), the curriculum in this area is housed within behavioral medicine (BM) and most primary care residencies make BM a strong curricular focus.^21^ BM is an interdisciplinary field that involves the integration of biological, psychological, and behavioral models of care.^22,23^ BM curricula usually focus on mental illness and addiction, counseling skills, the patient-physician relationship, and behavior change.^24^ For primary care residents, BM education has been shown to be most effective when there is an emphasis on the integration of new learning in the learners’ clinical context^25^ when it is interactive and ongoing.^26^ To our knowledge, the present study is the first to conduct an in-depth exploration of recent Canadian FM graduates’ experiences during residency training pertaining to MH.

Given the high burden of mental illness in Canada, the scarcity of specialty care, and the lack of literature exploring outcomes related to Canadian FM residency training in mental health, ***we sought to*** **understand whether FM residency curricula at the University of Toronto are adequately** ***preparing FP graduates to deliver MH care***. *To address this question, we* explored the experiences of recent FM residency graduates in providing primary mental health care, and their perceptions of mental health training gaps during their residencies.

## Methods

### Study design and participants

We conducted a qualitative descriptive study^27^ in tandem with thematic analysis^28,29^ to explore the experiences of recent FM residency graduates in providing mental health care, and their perceptions of residency training gaps, at the University of Toronto.^27^ A qualitative descriptive study design was used to address the study question and to provide a rich description of the context and process of completing the residency program and MH training from the participant's perspective while producing findings closer to the data.^27^ This study took place from December 2021 to April 2022 during the COVID-19 pandemic. Study participants consisted of recent graduates from the University of Toronto's Department of Family & Community Medicine's (DFCM) 24-month residency program. The DFCM spans 15 different teaching sites, which include urban sites located in downtown Toronto, suburban sites spread throughout the city and surrounding areas, as well as those located in rural communities north of the Greater Toronto Area. Using a purposive sampling method,^30^ potential participants were recruited via social media postings (eg, First Five Years Facebook page) and postgraduate Listserv. Only individuals who recently completed their FM residency at the University of Toronto (within the last 5 years), were eligible to participate. A total of ten participants were recruited for this study, with 2 participants not attending their scheduled interview. As a result, 8 individuals participated in semi-structured interviews ranging from 45 minutes to 1 hour, via Zoom, which were video and audio recorded. The PI (KL) designed the script for in-depth interviews based on a review of the literature identifying gaps in residency education of mental health care in Canada. The scripts were reviewed and edited by the other investigators (NB and AR) with input from faculty with expertise in qualitative methodology from the Office of Education Scholarship at the University of Toronto.

Questions probed participants’ experiences delivering mental health care and their perceptions of their learning needs. To our knowledge, no additional persons were present during the interviews besides the participants and researcher. Interviews consisted of 3 dyads and 2 one-on-one interviews, which were led by the research assistant, AR. Dyads were composed of individuals whose schedules were compatible, and to our knowledge, these individuals did not have pre-existing relationships. After each interview, field notes were taken by AR, to produce thick descriptions. As our research question seeks to understand whether the University of Toronto FM residency curricula adequately prepare FP graduates to deliver MH care by exploring graduate's residency experience and perception, dyadic interviews were chosen to allow both participants not only time to reflect and present their individual perspectives, but their shared, overlapping, and contrasting experiences.^31–33^ As such, dyadic interviews in conjunction with one-on-one interviews (ie, individual) were chosen for the following reasons: first, dyadic interviews were used to enable participants additional time to process information, and to stimulate experiential memories. Second, dyadic interviews enable the expansion of the topic of research beyond what might be possible with individual interviews. Third, unlike focus groups where there may be an overcrowding of voices and some individuals may be silenced, dyadic interviews limit this risk.^31–33^ Conversely, individual one-on-one interviews were used to allow participants to share their experiences and individual perspectives which may have been withheld in a public or shared context.^34,35^ Prior to interviews, participants completed a demographic survey (see Table 1, n = 7, response rate 88%) and provided consent as approved by the University of Toronto Research Ethics Board (REB). One survey was not completed and was subsequently omitted. The REB approved this study on December 7, 2021 (Ethics Approval Number 00041788). Verbal informed consent was also obtained from participants prior to the recording of interviews. Participants were informed of their right to withdraw from the study at any time. Once verbal consent was obtained, the recording of the interview commenced. Transcripts were de-identified and participants were referred to by their chosen pseudonyms. Due to time constraints, transcripts were not returned to participants for comments, and there were no repeat interviews. Each participant received a $50.00 gift card.

**Table 1.** Demographics of survey respondents and descriptive statistics (n = 7).

PARTICIPANTS CHARACTERISTICS	N	PERCENT
Average age		
< 29	4	57.1%
> 30	3	42.9%
Sex		
Male	4	57.1%
Female	3	42.9%
Residency sites		
THO MH	1	14.3%
North York General Hospital	2	28.6%
Mount Sinai Hospital	3	42.8%
Sunnybrook Hospital	1	14.3%
Years of independent practice		
< 1 year	1	14.3%
1 year	5	71.4%
> 3 years	1	14.3%
Location of practice		
Suburban	1	14.3%
Urban	6	85.7%
Type of current practice		
Group practice	6	85.7%
Solo practice	1	14.3%
Psychotherapy offered in practice		
Yes	2	28.6%
No	4	57.1%
Maybe	1	14.3%
Addiction management in practice		
Yes	1	14.3%
No	6	85.7%
Maybe	0	N/A

### Data analysis

**Figure 1.**
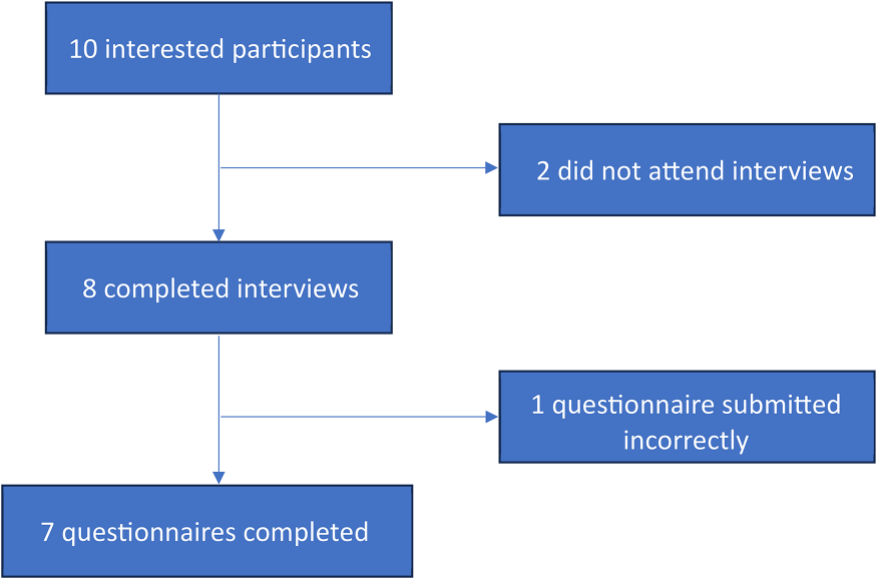
COnsolidated criteria for REporting Qualitative research (COREQ) flow diagram.

Descriptive statistics were performed on surveys (n = 7) and are presented in Table 1. One participant submitted a blank survey which was unable to be included in the descriptive analysis (see Figure 1).

Interviews were transcribed verbatim by the RA (AR). Each member of the research team (AR, NB, and KL) familiarized themselves with the data, and preliminary thematic analysis^31,32^ was performed by 2 team members (AR and KL) to develop an initial and evolving coding tree framework.^33^ Thematic analysis was used as a method to identify, analyze, and report patterns or themes of the data. Thematic analysis was also conducted within a constructivist orientation, as it is not linked to pre-existing theoretical or epistemological frameworks, enabling flexibility in the exploration and analysis of the data.^28,34^ Furthermore, this approach enabled us to explore how social, cultural, and structural contexts shape participant's experiences, knowledge, and meaning-making, constructed through interactions between the researcher and participant.^28,34^ Based on re-occurring data patterns, the team felt that thematic saturation had been achieved and no additional participants were recruited.^35^ Furthermore, in alignment with the findings discussed by Guest et al,^35^ majority of concepts discussed during interviews were presented by the first 5 to 6 participants. Utilizing NVivo 11 and Microsoft Excel, codes were queried to further develop, review, and define overarching themes and subthemes. Additionally, text segments within each code were further studied and analyzed in developing overarching themes.^36,37^

Both the PI (KL), and RA (AR) would meet regularly to discuss tensions, coding, identified themes in the data, and evolving coding tree framework. As the coding tree framework evolved, NB provided feedback to enrich the understanding of themes and conceptualization of the role of the FP, professional identity, curriculum renewal, and barriers in providing MH care. Using this inductive approach, we were able to refine the coding tree framework and identify potential areas for future research. Additionally, all research team members (AR, NB, and KL) engaged in peer debriefing, meeting several times to discuss ideas, assumptions, concepts, and meaningful patterns in the data to ensure validity. One team member (NB) served as an investigator triangulator and was not involved in data coding. This enabled triangulation of developing themes and concepts and cross-checking perspectives and assumptions. Such measures were taken in an effort to reduce intrinsic biases, prejudice, and preconceived perceptions/assumptions. In the study, rigor and trustworthiness were enhanced by using: (1) thick description, (2) peer debriefing, (3) audit trail and reflexivity, and (4) external audits.^38^

### Reflexivity service

Using a constructivist orientation, each member of the research team acknowledges how their experiences, perspectives, and positionality may shape the findings of this study. For example, AR is a doctoral student with an interest in professional identity formation among health professionals, and experience in conducting qualitative research. KL and NB are both FPs with training and a keen interest in mental health and addictions. Additionally, KL and NB both have experience in research related to mental health and addictions. KL is an Assistant Professor at the DFCM and a Postgraduate Site Director at a local teaching hospital. Her research explores access to care for patients with mental health concerns, educational scholarship in postgraduate medical education, and experiences of patients with mental health concerns in primary care. NB is a past program director of the DFCM Enhanced Skills in Addiction Medicine program. All members of the research team identify as females, with one being a visible minority. It is important to note, that as both KL and NB have leadership roles in the department, both members did not conduct interviews or have direct interaction with participants, in efforts to minimize potential power differentials or bias during interviews. Moreover, the interviewer (AR) did not have prior relationships or knowledge of any study participants. Throughout coding, team members met regularly to discuss how their own experiences, identities, and social location were impacting data analysis.

## Results

**Figure 2.**
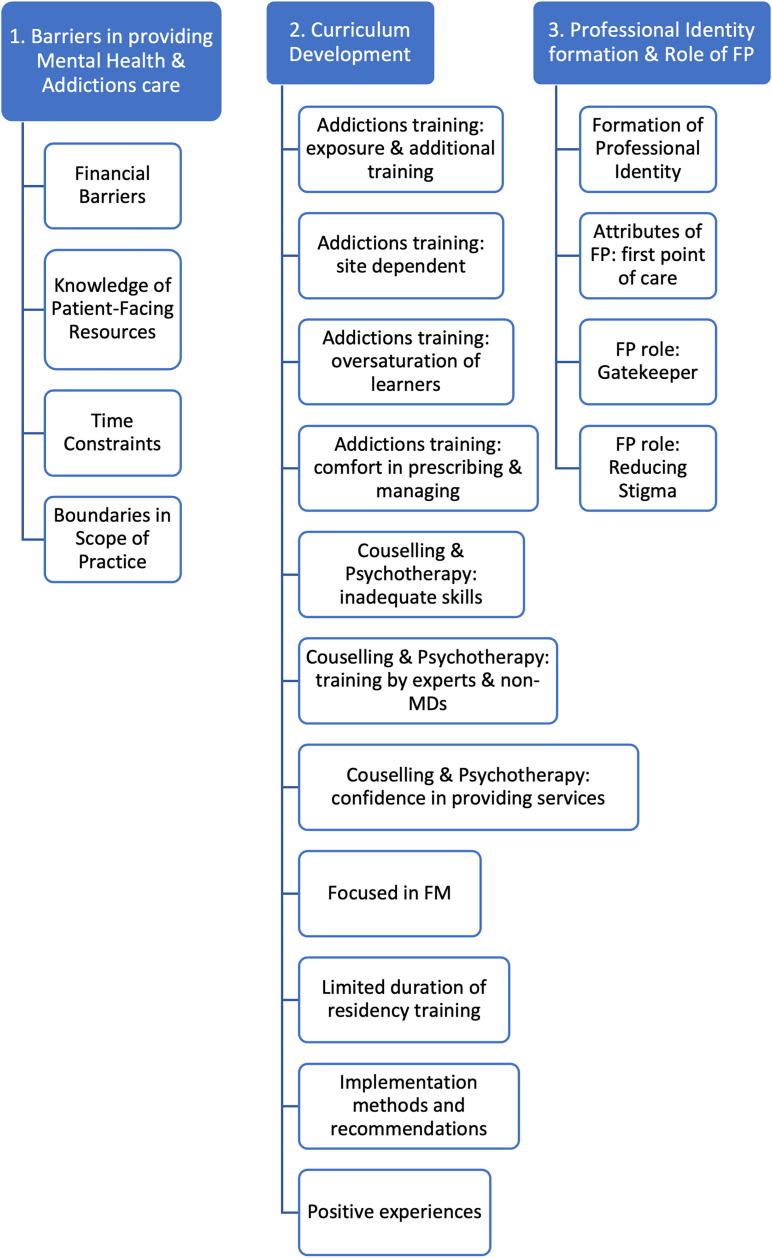
The 3 overarching themes and 19 significant subthemes developed during thematic analysis.

Three overarching themes and several sub-themes developed from the data (see Figure 2). The first, *Barriers in Providing Mental Health and Addictions Care*, encompassed several subthemes focusing on obstacles in providing MH care. The second theme, *Curriculum Renewal*, illuminated gaps within the FM residency curriculum that require further development. Lastly, the third theme, *Professional Identity and Role of Family Physician*, explored perceptions in ranges of clinical competency in providing MH care, formation of professional identity, and the role of the FP. For each subtheme, we provide representative quotes from the transcripts (see Table 2).

**Table 2.** Findings from interviews with recent family medicine resident graduates.

THEME	SUB-THEME	ILLUSTRATIVE QUOTATIONS FROM RESIDENT GRADUATES
1. Barriers in providing Mental Health and Addictions Care	**Financial Barriers**	There are additional training resources for us to take training session on psychotherapy and such, but unfortunately that's not part of residency, and once you finish residency, those kind of training cost money, and you have to think about it and say practitioner do you want to invest the time and money in doing the psychotherapy.—Ling
	**Knowledge of Patient-Facing Resources**	… it's very easy to tell patients why don’t you just look up this website or whatever, it's very different when you’re actually counseling them how to do that, or what else that they are going to be doing at the same time. And that's, I think that's where the burden of primary care is coming.—Sarah
	**Time Constraints**	I think what family doctors do lack in terms of mental health patient is time. We obviously don’t have an hour to spend with mental health patients. And every single time like psychiatrist may. And so it sometimes challenging to effectively treat mental health patients “cause the amount of time that's involved.”—Scott
	**Boundaries in Scope of Practice**	…You enter practice [and get] a lot of questions like…I think I have OCD, I think I have ADHD … I wish I could give people a bit of a clear answer I feel like comfortable getting things started. Cause there can often be a really long wait for these assessments … I wish that not necessarily that I would be the sole person managing it, “cause I don’t think that's sort of my scope.”—Jeff
2. Curriculum Renewal	***Addictions Training:* Lack of exposure and seeking additional training**	I didn’t see as much addiction medicine in these areas during my residency … so I would say my comfort level for certain addictions [is] not as great, and it (my comfort) is simply based on the exposure I’ve had in residency.—Kimberly
	***Addictions Training:* Site Dependent**	I felt like I got very little exposure through the standard curriculum. Maybe part of it was the site that I was at, like it was less common. In hospital for example there weren’t really a lot of people that were admitted for a substance issue that saw Psychiatry.—Jeff
	***Addictions Training:* Oversaturation of learners**	[…] These clinics were only a few times a month and so residents would have to be split you know share the time. And it ended up being out of the five family medicine blocks that we do in residency in a year, for example in the first year, maybe each resident would get to work in the clinic twice a year, so it was limited time unfortunately, but it's a great resource for learning.—Kimberly
	***Addictions Training:* Comfort in prescribing and managing**	[…] I think it depends on the substance we are talking about. I think of in terms of nicotine, and alcohol I’m very well trained and is a very common place but if we’re talking about something a bit more hardcore, like cocaine addiction, meth addiction, then unfortunately I don’t feel like I had enough exposure. I tend to just refer those patients to addiction services.—Ling
	***Counselling-Psychotherapy Training: Defining counselling or therapy***	I guess that depends on what you label as counselling services or psychotherapy. I’m happy to follow-up with my patients every two weeks or whatever just to talk and make goals and things. So, in that case I guess you would consider it counseling. I do a lot of that, mind you I’m very interested in this type of thing. Many of my colleagues in the same clinic don’t do that. They would just you know refer to bounce-back or you know psychotherapy or one link or something like that.—Sarah
	***Counselling-Psychotherapy Training:* Additional training-inadequate skills**	I think the prevalence as well, 1 in 4 people will have a mental health problem, it's kind of like saying you don’t know how to treat diabetes as a family doctor, that the same you know as saying I don’t know what to do with mental health. Uhm, yah I think that's not acceptable. And I think they need to do more to help residency acquire the skills.—Jaylin
	***Counselling-Psychotherapy Training:* Training by experts and non-MDs**	[…] We had several amazing social workers in our family health teams who were a wealth of knowledge and a wealth of resources and taught us so much about where to refer to and what resources are available in the GTA. And I think that helped quite a bit with my comfort level for my addictions patients.—Scott
	***Counselling-Psychotherapy Training:* Confidence in providing services**	I personally don’t do a lot of counselling myself. I mean part of the reason is truthful, is I don’t feel like I have the skills to be doing it.—Roy
	**Training Should be Specific to Family Medicine**	I think also emphasising to if you are doing a like a Psychiatry rotation or having non-family physicians’ preceptors as part of the curriculum, it's just emphasising that these are family physicians’ residents. So whatever training whatever knowledge they want to impart should be from a family physicians’ lens. So, what family docs see most, what would be most useful for their practice.—Scott
	**Limited duration of residency training**	With a two-year residency I felt like it just wasn’t enough to give me the expertise that I wanted in the area that I want to practice.—Dwight
	**Implementation Methods and recommendations**	See Table 3
	**Positive Experiences**	I think our family residency trained us pretty well for at least the common presentations. We had some quite a few patients who presented with depression and anxiety. We had quite about of time for counseling. We had supervisions for those sessions. We had dedicated teaching for those sessions. So at the end of the day I think it comes down to using that experience using those skills and practising them, which we had an advantage for because our longitudinal curriculum.—Scott
3. Professional Identity formation and Role of the Family Physician	**Formation of Professional Identity**	Some people might actually think that it's not a physician's role to have those types of skills. But I really think that you know, at least having some bedside skills, at least an understanding of those therapies can be useful.—Roy
	***Family Physician's Role:* Attributes of Family Physician's: First point of care**	…And I think in family medicine that we’re seen as the first point of care, so you have to be able to have that kind of patience as well as make a plan and make goals and meet back and really talk to patients. So, patience is one of them. Being empathetic is another one, and I think being that leader, you know being the person who can, not necessarily cheer-lead the patient on, but engage the patient in motivational conversation, at the same time in recognizing and being prepared to give feedback when you need and to support patients.—Sarah
	***Family Physician's Role:* Gatekeeper**	Because our mental health system let's say in the province or even in the GTA, can kind of be convoluted for some patients to navigate, we can kind of be a bit like a gatekeeper, but kind of like more of a steward of the resources, pointing patients in the right direction…—Roy
	***Family Physician's Role:* Reducing Stigma**	The connections you have with your patients, these are the people you are going to have long-term relationships. You’re going to be their doctor for years and even decades right. So, I think because we are the ones looking after them first, it's kind of already builds on the trust and the relationship we already have, and I think people are more likely to open up about their mental health conditions. And I also feel like it's less shameful because I know some patients are like “oh I don’t want to see a psychiatrist” because of the stigma associated with that.—Jaylin

### Theme 1: Barriers in providing mental health and addictions care

Common barriers to providing mental health care in primary care included financial barriers, knowledge of patient-facing resources, time constraints, and scope of practice boundaries. These factors combined often precluded FP from providing comprehensive mental health and addiction care to patients.

#### Financial barriers

Many FPs reported financial barriers to undertaking additional optional training to supplement their MH knowledge and skills, resulting in feeling ill-equipped to deal with some of the day-to-day presentations they may encounter. Consequently, a lack of affordable continuing professional development training may have negative downstream effects on patient care and continuity of care, as FPs are unable to upscale and enhance their MH knowledge and skills.

#### Knowledge of patient-facing resources

Some participants expressed difficulty accessing mental health resources when transitioning from residency to practice. This was a barrier to providing day-to-day mental health care, as physicians often felt inadequately prepared to empower patients in their own care.

#### Time constraints

Most participants reported that, due to time constraints, they were often unable to offer adequate mental health services. This barrier was frequently coupled with competing physical health issues. As a result, physicians found themselves navigating the tension of dedicating time to physical health issues which took time away from attending to mental health issues.

#### Boundaries in the scope of practice

Some participants felt unprepared to effectively treat certain mental health concerns in primary care, due to limitations in their own level of expertise or scope of practice. When their patients’ mental health issues were beyond their scope of practice or expertise, many reported referring patients to more specialized care (ie, psychiatry and addiction medicine).

### Theme 2: Curriculum renewal

This theme highlights the need to develop certain areas within the FM residency curriculum, and how current gaps are sometimes perceived as barriers in providing mental health care among recent FM graduates.

#### Addictions training

Most participants recommended increased curriculum development in substance use and addiction. Many believed that a lack of exposure to patients living with substance use disorders contributed to their overall discomfort caring for this population. Participants reported referring to more specialized services, therefore prolonging patients’ waiting time for treatment. Participants also felt they had to seek additional training beyond their FM residency to feel comfortable caring for these patients, citing differences in exposure among training sites and a lack of FM preceptor role models delivering substance use care as common reasons for unpreparedness.

#### Counselling-psychotherapy training

The majority of participants expressed that all FPs should provide some counseling/therapy, however, many felt that “*psychotherapy*” is not well-defined and was not adequately taught during residency. Additionally, uncertainties existed regarding the scope of practice, for example, what FPs should be able to offer versus specialized care providers such as social workers, etc. Many participants also highlighted the training they received from allied health professionals (eg, social workers) in psychotherapy and/or counseling skills was vital to their education. However, most did not feel confident in providing counseling/psychotherapy services.

#### Training should be specific to FM

Most participants agreed that training during residency in mental health should be rooted in the specific learning needs of FPs. Teaching from non-FM physicians is often too specialized or not relevant to FM needs and context.

#### Limited duration of residency training

As the FM residency program is currently limited to 2 years, many participants did not feel comfortable providing more in-depth care to populations requiring additional specialized care.

#### Implementation methods

Participants identified curriculum implementation methods that were successful in delivering content and addressing current implementation needs (see Table 3). For example, the majority of participants highlighted the positive ways in which their residency training prepared them and the need for a mental health curriculum to be delivered longitudinally. Moreover, some participants also expressed the need for increased resources when transitioning in and out of residency.

**Table 3.** Implementation methods and recommendations from theme #3: Curriculum renewal.

SUB-THEME	CATEGORIES	ILLUSTRATIVE QUOTATIONS FROM RESIDENT GRADUATES
Implementation Methods	**Transitioning to residency**	[…] you know just like when you start your general rotations, or rotations that have a large breadth of knowledge, they do a little bit of a bootcamp at the beginning, I think that can really help with psychiatry, mental health, and addictions. Like before people kind of start their rotation, in the transition to residency period, just like a little bit of a bootcamp where everyone gets on the same page of base knowledge that we need.—Roy
	**Curriculum Development: Longitudinal approach**	[…] A lot of times for interventions take time to actually come into effect. And so, seeing a patient once or twice in a month, is I don’t think is as effective as following that patient for months or years to actually see the benefits of your counseling or the benefits of the medications you’ve prescribed uhm so with whatever curricula methods ends up being developed I think a longitudinal method makes more sense.—Scott
	**Curriculum Development: Serving diverse population needs**	I actually feel like I had very little exposure to doing mental health for ethnic minority. I didn’t really have a whole lot of encounters with those types of patients. And I think they have specific mental health needs that are not quite the same as the mainstream. So, I didn’t really have a lot of immigrant or refugee patients or people of colour in general.—Ling
	***Curriculum Implementation:* Mixture of didactic and hands-on experience**	… I definitely think some sort of practical training, some sort of one-on-one or like in small groups for example … I think a variety will always be better. And maybe hearing from interprofessional providers as well … like resources available including sessions with like a social worker from the family health team for example, to get a different perspective as well.—Jeff …some residents they thrive in a didactic setting … recently residents have been looking for more in the moment teaching, [which] would be like in rotations or sometimes in role play… I would try and be mindful of the different types of learners and try … develop a curriculum that that can be used and benefit from, from a variety of different learning styles. [Having] a devoted core rotation in psychiatry, in mental health and addiction is ultimately beneficial.—Dwight
	***Curriculum Implementation:* Exposure**	…despite getting didactic teaching and even an OSCE simulation I feel it wasn’t enough for me to comfortably manage it on my own and I would be looking to refer to a specialist or opioid use clinic for example.—Kimberly
	***Learning Improvements*: Resources**	I would definitely have some teachings on community resources. Teaching the residents about the tools and referral services that are there at their disposal, so not just limiting themselves to skills, but arming them with the skills to ask for additional help.—Ling Maybe in residency learning more about the resources available OHIP vs non-OHIP might be helpful in transitioning into staff.—Kimberly

#### Transition to residency

This subtheme encompassed discussions about curriculum development, positive learning experiences, and how certain aspects of curriculum implementation and delivery can be improved. To ensure all residents have a foundational base knowledge, one participant suggested that the implementation of a transitionary phase or “bootcamp” will help augment gaps in knowledge.

#### Longitudinal approach

Another re-occurring discussion surrounding curriculum development was the optimal format for curriculum delivery. Many participants reflected on residency projects, didactic teaching, the use of observed structured clinical exams (OSCEs), and academic half-days dedicated to mental health, which all aided in their learning. However, others advocated for delivering mental health curricula longitudinally. While some participants acknowledged that not all residency sites offered a longitudinal curriculum, participants highlighted the importance of practicing certain psychotherapy and counseling skills (eg, CBT), and being able to witness the outcomes of treating patients with mental health issues over time.

#### Serving diverse population needs

Many participants expressed that differences in training were often site-dependent. Reflecting on the service needs of mental health patients, one participant highlighted the need for increased exposure in treating mental health issues among ethnic minority patients and other underrepresented groups.

#### Resources

Several participants indicated how useful resources provided during residency are in providing patient care postgraduation. Moreover, residents also believed that additional support and resources should be available in assisting residents’ transition from residency to practice. One participant went on to suggest the DFCM adopt a centralized online platform where mental health resources are easily accessible.

#### Positive experiences

Although much of the discussion among participants focused on areas for improvement within the curriculum, there were several positive experiences that were highlighted by participants. These included residents receiving feedback on counseling skills and receiving training from allied health professionals such as social workers who imparted their skills and expertise. Overall, many participants expressed that they felt residency training prepared them for dealing with many of the most common mental health presentations (ie, depression and anxiety).

### Theme 3: Professional identity formation and role of the FP

#### Formation of professional identity

Although participants agree that FPs should be equipped with the skills to treat common mental health presentations, many FPs perceived that it is not within their scope to provide psychotherapy or counseling. This perception influences how FPs view what acts of service fall within their scope of practice, which therein impacts the formation of their professional identity.

#### FP's role

All participants commented on the importance and unique role of FPs in providing mental health care. In addition to participants identifying FPs as the “gatekeepers” to healthcare resources, many acknowledged FPs as the first point of care. They highlighted the distinct relationship between FPs and their patients that uniquely positions FPs to provide mental health care within the mutual trust built through a longitudinal relationship.

#### Attributes of FPs/first point of care

Common attributes participants perceived FPs to have that make them well suited to provide mental health care to patients included *empathy, being supportive, and helping patients set goals.* Moreover, FPs are often the “first boots on the ground,” which positions them as the first providers to address mental health concerns. As the first point of care, many participants emphasized the importance of adequately training FPs to be able to address patients presenting with mental health concerns.

#### Gatekeeper

When describing the role of an FP in providing mental health care, some participants used the analogy of a “gatekeeper” of healthcare resources while helping patients navigate the system and connecting them to the appropriate care needs. This perception positions FPs in dual roles, both as advocates for patients and stewards of resources.

#### Reducing stigma

The longitudinal relationship between FPs and patients helped to break down feelings of shame and stigma associated with seeking mental health care. Patients often felt more comfortable stating they were seeing their family doctor for an issue rather than a psychiatrist.

## Discussion

Using Thematic Analysis within a constructivist framework, we explored the reflections of recent FM residency graduates on their training in MH during their residencies. Echoing prior findings and the *Outcomes of Training* project report by the CFPC (2022), our study demonstrates that present training in MH is highly variable, lacking in certain areas, and supports the CFPC's call for curriculum renewal in this area.^18^ This was highlighted through the 3 themes that permeated our study: (1) barriers in Providing MH Care, (2) curriculum renewal, and (3) professional identity formation and the role of the FP.

In our study, participants attributed insufficient MH training to a lack of exposure and inclusion in the formal curriculum. Although some participants sought MH training via electives on their own accord, most participants perceived their training specifically in the areas of psychotherapy and addiction to be inadequate, impacting their overall confidence and comfort in providing these services and caring for these patients.^6,39,40^ Our data also purport that differences among training sites impacted overall learning outcomes, which included both an oversaturation of learners at some training sites and limited exposure to marginalized populations. Furthermore, many of our study participants reiterated the findings of previous research highlighting the need for longitudinal curriculum delivery centered in FM.^41–43^ Additionally, participants expressed that although they received training in MH care, it was often too specialized and not centered on FM or did not reflect the needs of primary care.^44^

**Figure 3.**
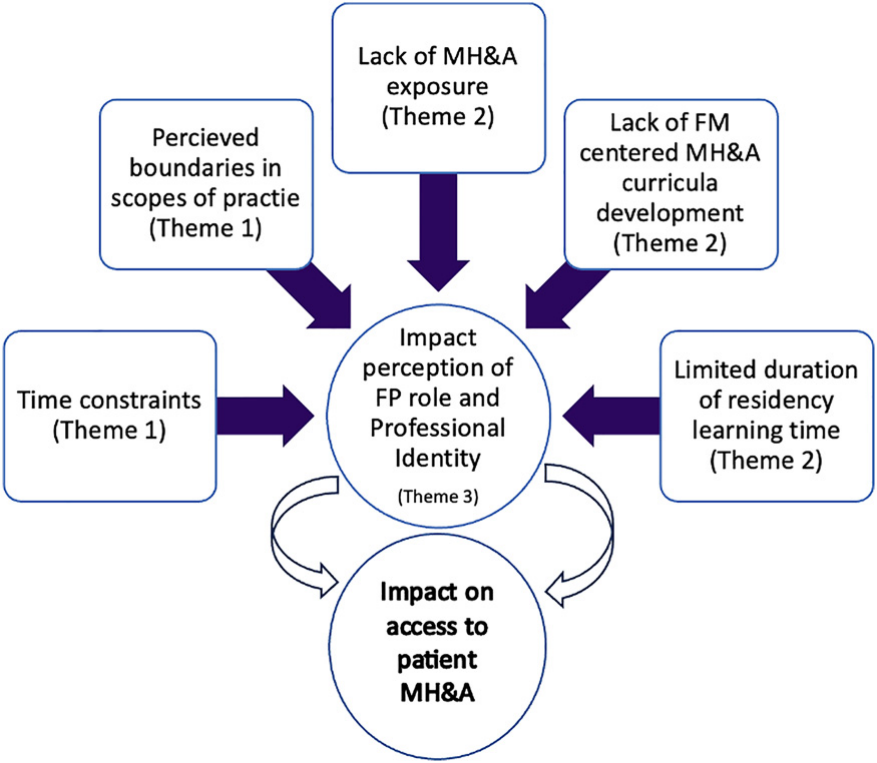
Our findings suggest that access to mental health care by patients may be hampered by a host of factors which include: clinical time constraints, perceived boundaries in scopes of practice, inadequate exposure to mental health and addiction (MH&A) presentations during residency training, lack of MH&A curricula centered on the needs of family medicine, and limited duration of residency and learning time. Furthermore, when combined, these factors may have a compounding impact on an FP's perception of their role and professional identity, which subsequently may impact patient access to MH&A care.

The concept of professional identity was seen to be a fundamental common thread among participants’ experiences (see Figure 3). As residents learn and adopt a set of values, behaviors, and norms of the profession during residency training through socialization, their self-efficacy is rooted in their positive experiences during training, which enables them to think, act, and feel like physicians.^45,46^ In doing so, they develop their own conceptualization of what it means to be an FP. Bandura's Social Learning Theory (1977)^47^ explains this well; if residents are not adequately exposed to an area of focus during their training, or it is not role modeled well or shown to be an essential part of the FP's role, then residents will not have the intrinsic motivation or self-efficacy to provide care in this area when they enter practice. In turn, this becomes a feedback mechanism impacting FP's perceptions and comfort in managing MH concerns, underlining the need for further curricular development in this area.^46^ As such, residency programs must ensure that the educational journeys of residents, which include formal, informal, and hidden curricular experiences, balance the expectations, experiences, and professional goals of residents to foster one's professional identity in this specific area of training.^48^

In addition to professional identity, many participants reflected on the role of the FP in relation to providing MH care. Consistent with previous research, participants indicated that FPs play a significant role in providing MH services, are often patient's first point of contact, and are seen as gatekeepers to healthcare resources.^49–52^ Participants also remarked on the unique patient-provider relationship FPs build longitudinally. Building on previous research, our findings illustrate FPs’ commitment to provide longitudinal comprehensive care that is centered on the patient-physician relationship.^53^ Factors that were attributed to FPs role, uniquely situating them to provide MH care included: patients able to access care in a low-barrier, low-stigmatizing environment^54–56^ and FPs longitudinal patient-provider relationship.^52,56^ Furthermore, the most common presentations participants reported to have encountered in their practices were depression or anxiety, in alignment with existing literature.^14,57^ Consistent with recent findings, our study reflects that although FPs are heralded as gatekeepers to the healthcare system, they continue to feel discomfort in identifying and managing less common presentations such as ADHD or stimulant use disorder.^58^ We argue that feelings of discomfort may be related to boundaries in scopes of practice, as participants reported referring these patients on to more specialized care (ie, psychiatry and addiction medicine) due to discomfort in managing them within primary care. Additionally, given the shortage of mental health specialists in Canada and long wait times to be seen by psychiatry,^12^ this highlights the importance of training FM residents who are confident in treating more complex MH presentations.

In the US, integrated BM curricula are the norm in primary care residencies.^24^ Residents who trained in a program with a strong foundation in BM felt more confident treating behavioral health conditions.^59^ Integrating BM within primary care has been shown to reduce the severity of common mental health disorders and enhance patients’ experience of care.^60^ By weaving BM through the residency curriculum, residency programs will train residents to develop strong professional identifies as FPs who recognize the importance of treating the whole person using a biopsychosocial lens. Curriculum in this area should include a component of interactivity, that is, role play, iterative, and rooted in theory, utilizing lessons learned from integrative BM curricula that have shown success in many US primary care residency programs.^26^

Despite many participants’ best efforts to provide MH care, there were several barriers identified by participants. These barriers included financial barriers in acquiring additional MH & addiction training, financial disincentives that deter treatment for MH conditions in current payment models,^62^ limited access to patient-facing resources,^61^ time constraints, and perception of the scope of practice boundaries.^62,63^

## Implications for FM Residency Education

This study identified the need for curriculum renewal in MH to bolster the current FM residency to equip future FPs with the skills and tools necessary to provide evidence-based, comprehensive care for patients with MH concerns. The goal of this study was to highlight the impact and tensions recent FP graduates are experiencing due to insufficient formal learning opportunities. Both formal and hidden curricula have a central role in the professional identity formation and FP's perception of the role they play in the greater healthcare landscape.

## Study Limitations

There were several study limitations. Our sample consisted of 8 participants and included only recent graduates of the University of Toronto's Family Medicine Residency Program. Our demographic survey response rate was 88% (n = 7). Survey and interview guide (Appendix 1) questions were not verified or used in a pilot study. Therefore, our findings may not be generalizable to the experiences of all FM residents across Canada or in other countries. It is worth noting, however, that a sample of this size can often be seen in qualitative research, and that the achievement of data saturation was prioritized in this study instead of reaching a certain sample size.^64^ There may also be a selection bias in this study due to sample composition. Most of the individuals interviewed had a particular interest and/or focused practice in MH, and many had sought out additional training in this area after residency completion. All interviews were conducted online over Zoom rather than in person due to pandemic restrictions. This can lead the interviewer to potentially miss subtle nonverbal cues that may have been identified had the interviews taken place in person. The original intention was to hold focus groups, however, due to scheduling challenges on the part of the participants, the interviews took place as either single interviews or dyads. Additionally, this study did not explore the diagnosis and treatment of ethnic minorities and underrepresented groups. Furthermore, the findings of the study were not made available to the participants of the study prior to publication for their feedback.

## Future Directions

As this study only looked at a small sample size of recent graduates from the University of Toronto's FM residency program, it would be important to conduct further qualitative research with recent graduates from FM programs across Canada. Future studies should explore the diagnosis and treatment of MH conditions in ethnic minorities and underrepresented groups and whether residency education needs to be adapted when addressing these patient populations. Finally, although addressed briefly in this study, the focus on addiction training during FM residency was not explored in detail. Additional research should focus specifically on residents’ experiences with addiction training during residency.

## Conclusion

FPs play a pivotal role in caring for patients with MH concerns. Participants identified a number of barriers to optimal learning specific to mental health and addiction during their residencies. Opportunities to enhance the FM residency curriculum should be undertaken to increase exposure, and improve FM-centered MH teaching that is longitudinal, includes a mix of didactic and hands-on experiences, and clearly defines boundaries in the scope of practice in primary care. Attention must be paid to transitions in and out of residency to support resident self-efficacy, knowledge of community resources, and learning enhancements specifically in the areas of addiction care and psychotherapy, and counseling. This will enable residents to develop a strong professional identity where evidence-based, comprehensive, patient-centered MH care does justice to the future of FM in Canada.

## Supplemental Material

sj-docx-1-mde-10.1177_23821205241238642 - Supplemental material for “Not doing it justice”: Perspectives of Recent Family Medicine Graduates on Mental Health and Addictions Training in Residency Supplemental material, sj-docx-1-mde-10.1177_23821205241238642 for “Not doing it justice”: Perspectives of Recent Family Medicine Graduates on Mental Health and Addictions Training in Residency by Abigail Ramdawar, Nikki Bozinoff and Kimberly Lazare in Journal of Medical Education and Curricular Development

sj-pdf-2-mde-10.1177_23821205241238642 - Supplemental material for “Not doing it justice”: Perspectives of Recent Family Medicine Graduates on Mental Health and Addictions Training in Residency Supplemental material, sj-pdf-2-mde-10.1177_23821205241238642 for “Not doing it justice”: Perspectives of Recent Family Medicine Graduates on Mental Health and Addictions Training in Residency by Abigail Ramdawar, Nikki Bozinoff and Kimberly Lazare in Journal of Medical Education and Curricular Development

sj-pdf-3-mde-10.1177_23821205241238642 - Supplemental material for “Not doing it justice”: Perspectives of Recent Family Medicine Graduates on Mental Health and Addictions Training in Residency Supplemental material, sj-pdf-3-mde-10.1177_23821205241238642 for “Not doing it justice”: Perspectives of Recent Family Medicine Graduates on Mental Health and Addictions Training in Residency by Abigail Ramdawar, Nikki Bozinoff and Kimberly Lazare in Journal of Medical Education and Curricular Development

sj-pdf-4-mde-10.1177_23821205241238642 - Supplemental material for “Not doing it justice”: Perspectives of Recent Family Medicine Graduates on Mental Health and Addictions Training in Residency Supplemental material, sj-pdf-4-mde-10.1177_23821205241238642 for “Not doing it justice”: Perspectives of Recent Family Medicine Graduates on Mental Health and Addictions Training in Residency by Abigail Ramdawar, Nikki Bozinoff and Kimberly Lazare in Journal of Medical Education and Curricular Development

## Appendix 1

### Semi-structured Interview Guide

Reference to *“mental health” also* includes addiction.

1. What was your experience with mental health training during your residency?
  - What worked well?
  - Is there anything that did not work well?
2. What do you see as the role(s) of family physicians in attending to mental health issues? What skills/qualities do family physicians have that make us well-positioned to deal with the mental health issues of our patients?
  - What skills/qualities do we not have as family physicians to deal with these issues?
3. What are the most common mental health presentations you are currently seeing in your practice?
  - Do you feel your residency training adequately prepared you to deal with these common presentations?
  - What was most helpful in preparing you, what would you like to have done differently?
  - How do you feel the COVID-19 pandemic has impacted your patients’ mental health and your ability to deliver care related to mental health issues?
4. - Tell me, how comfortable are you with dealing with addiction-related issues?
  - Given the opioid crisis, what is your comfort in medication-assisted treatment of opioid use disorder, and do you feel your training prepared you to deal with this?
5. With respect to counseling skills and/or psychotherapy, did you receive any training during your residency? If so, what was the training about/ like?
  - Tell me, did you receive any training around counselling skills (i.e., motivational interviewing, solution-focused therapy, Cognitive Behavioural Therapy (CBT), etc.) during your residency?
  - With respect to the training you received, was it one-on-one supervised training or didactic teaching?
6. Do you offer more structured counseling and/or psychotherapy services as part of your practice?
  - If so, how is it going?
  - If not, are there reasons as to why you do not offer these services?
7. Now that you are in practice, are there any resources you would have found helpful during your residency training regarding mental health and addiction teaching?
  - Tell me, is there anything you believe is missing with respect to mental health and addiction training?
8. If you were to design content for a mental health curriculum for incoming family medicine residents at the University of Toronto, what would that curriculum ideally look like? How would it ideally be delivered?
