# Supplementary material for: “Not doing it justice”: Perspectives of Recent Family Medicine Graduates on Mental Health and Addictions Training in Residency
Source: J Med Educ Curric Dev. 2024 Apr 9;11:23821205241238642. doi: 10.1177/23821205241238642 (PMC11005487; doi:10.1177/23821205241238642)
Supplement: sj-docx-1-mde-10.1177_23821205241238642 - Supplemental material for “Not doing it justice”: Perspectives of Recent Family Medicine Graduates on Mental Health and Addictions Training in Residency [file sj-docx-1-mde-10.1177_23821205241238642.docx]

**Appendix 1:**

**Semi-structured Interview guide**

Reference to *‘mental health’ also* includes addiction.

1. What was your experience with mental health training during your residency?
   1. What worked well?
   2. Is there anything that did not work well?

1. What do you see as the role(s) of family physicians in attending to mental health issues? What skills/qualities do family physicians have that make us well-positioned to deal with mental health issues of our patients?
   1. What skills/qualities do we not have as family physicians to deal with these issues?

1. What are the most common mental health presentations you are currently seeing in your practice?
   1. Do you feel your residency training adequately prepared you to deal with these common presentations?
   2. What was most helpful in preparing you, what would you like to have done differently?
   3. How do you feel the COVID-19 pandemic has impacted your patients’ mental health and your ability to deliver care related to mental health issues?
2. 1. Tell me, how comfortable are you with dealing with addiction related issues?
   2. Given the opioid crisis, what is your comfort in medication-assisted treatment of opioid use disorder, and do you feel your training prepared you to deal with this?

1. With respect to counselling skills and/or psychotherapy, did you receive any training during your residency? If so, what was the training about/ like?
   1. Tell me, did you receive any training around counselling skills (i.e., motivational interviewing, solution-focused therapy, Cognitive Behavioural Therapy (CBT), etc.) during your residency?
   2. With respect to the training you received, was it one-on-one supervised training or didactic teaching?

1. Do you offer more structured counselling and/or psychotherapy services as part of your practice?
2. If so, how is it going?
3. If not, are there reasons as to why you do not offer these services?

1. Now that you are in practice, are there any resources you would have found helpful during your residency training regarding mental health and addiction teaching?
2. Tell me, is there anything you believe is missing with respect to mental health and addiction training?

1. If you were to design content for a mental health curriculum for incoming family medicine residents at the University of Toronto, what would that curriculum ideally look like? How would it ideally be delivered?
