# Supplementary material for: “Not doing it justice”: Perspectives of Recent Family Medicine Graduates on Mental Health and Addictions Training in Residency
Source: J Med Educ Curric Dev. 2024 Apr 9;11:23821205241238642. doi: 10.1177/23821205241238642 (PMC11005487; doi:10.1177/23821205241238642)
Supplement: sj-pdf-3-mde-10.1177_23821205241238642 - Supplemental material for “Not doing it justice”: Perspectives of Recent Family Medicine Graduates on Mental Health and Addictions Training in Residency [file sj-pdf-3-mde-10.1177_23821205241238642.pdf]

| Name | Files | References | Created On | Created... | Modified On | Modified By | Color |
|---|---|---|---|---|---|---|---|
| 1. Barriers in Providing Me... | 5 | 17 | 2022-08-07, 12:40... | EAR | 2022-08-07, 12:50.. | EAR | ● |
| Addressing Knowledge... | 8 | 19 | 2022-08-07, 12:49... | EAR | 2022-08-09, 10:12.. | EAR | |
| Sought out extra trai... | 8 | 31 | 2022-08-09, 10:12... | EAR | 2022-07-03, 11:22.. | EAR | |
| Boundaries in Scope of... | 4 | 26 | 2022-08-07, 12:49... | EAR | 2022-09-26, 9:25... | EAR | |
| Financial Barriers | 2 | 2 | 2022-08-07, 12:44... | EAR | 2022-08-07, 10:07.. | EAR | |
| Knowledge of Patient F... | 8 | 24 | 2022-08-07, 12:49... | EAR | 2022-08-07, 12:49.. | EAR | |
| Time Constraints | 5 | 15 | 2022-08-07, 12:49... | EAR | 2022-08-07, 10:03.. | EAR | |
| 2. COVID-19 | 6 | 19 | 2022-08-07, 12:40... | EAR | 2022-08-07, 12:41.. | EAR | ● |
| Impact on Patient Care | 9 | 21 | 2022-08-07, 2:16 PM | EAR | 2022-08-07, 2:17 PI | EAR | |
| Impact on Quality of Tr... | 3 | 4 | 2022-08-07, 2:17 PM | EAR | 2022-08-07, 2:17 PI | EAR | |
| Virtual Care | 2 | 4 | 2022-08-07, 2:16 PM | EAR | 2022-08-07, 2:16 PI | EAR | |
| 3. Curriculum Development | 8 | 51 | 2022-08-07, 12:41... | EAR | 2022-08-07, 12:41.. | EAR | ● |
| Addictions Training | 9 | 77 | 2022-08-07, 2:25... | EAR | 2022-08-07, 3:22... | EAR | |
| Centered in Family Med... | 7 | 20 | 2022-08-07, 2:26... | EAR | 2022-08-07, 10:03.. | EAR | |
| Counselling-Psychothe... | 9 | 94 | 2022-08-07, 2:26... | EAR | 2022-08-07, 10:34.. | EAR | |
| Implementation Methods | 3 | 10 | 2022-08-07, 2:39... | EAR | 2022-08-07, 2:40... | EAR | |
| Learning Improvement | 9 | 83 | 2022-08-07, 2:41 PM | EAR | 2022-08-07, 2:41 PI | EAR | |
| Longitudinal | 4 | 9 | 2022-08-07, 2:40... | EAR | 2022-08-07, 2:40... | EAR | |
| Positive Learning Ex... | 4 | 12 | 2022-08-07, 2:43... | EAR | 2022-08-07, 2:43... | EAR | |
| Transitionary phases | 3 | 7 | 2022-08-07, 2:42... | EAR | 2022-08-07, 2:43... | EAR | |
| Limited duration of resi... | 4 | 7 | 2022-08-07, 2:28... | EAR | 2022-08-07, 2:29... | EAR | |
| 4. Professinoal Identity &... | 4 | 23 | 2022-08-07, 12:41... | EAR | 2022-08-07, 12:41.. | EAR | ● |
| Expert-Expertise | 4 | 39 | 2022-08-07, 2:43... | EAR | 2022-08-09, 9:32... | EAR | |
| Common Presentation | 7 | 28 | 2022-08-07, 10:08... | EAR | 2022-08-07, 10:09.. | EAR | ● |

| | | | | | |
|---|---|---|---|---|---|
| Personal Interest | 3 | 7 | 2022-08-09, 9:32... | EAR | 2022-06-15, 8:43... |
| Family Physician role-q... | 6 | 22 | 2022-08-07, 2:45... | EAR | 2022-08-07, 2:46... |
| Relationship betwee... | 3 | 4 | 2022-08-07, 2:46... | EAR | 2022-08-07, 2:46... |
| Stigma and acces... | 3 | 3 | 2022-08-07, 2:46... | EAR | 2022-08-07, 2:47... |
| Formation of Professio... | 5 | 19 | 2022-08-07, 2:44... | EAR | 2022-08-07, 2:45... |
| Ranges of clinical com... | 6 | 33 | 2022-08-07, 2:43... | EAR | 2022-08-07, 2:44... |

EAR
EAR
EAR
EAR
EAR
EAR
