# Supplementary material for: “Not doing it justice”: Perspectives of Recent Family Medicine Graduates on Mental Health and Addictions Training in Residency
Source: J Med Educ Curric Dev. 2024 Apr 9;11:23821205241238642. doi: 10.1177/23821205241238642 (PMC11005487; doi:10.1177/23821205241238642)
Supplement: sj-pdf-4-mde-10.1177_23821205241238642 - Supplemental material for “Not doing it justice”: Perspectives of Recent Family Medicine Graduates on Mental Health and Addictions Training in Residency [file sj-pdf-4-mde-10.1177_23821205241238642.pdf]

Name		Files	References	Created On	Created...	Modified On
1. Barriers in Providing Me...		5	17	2022-08-07, 12:40...	EAR	2022-08-07, 12:50..
Addressing Knowledge...		8	19	2022-08-07, 12:49...	EAR	2022-08-09, 10:12..
Sought out extra trai...		8	31	2022-08-09, 10:12...	EAR	2022-07-03, 11:22..
Boundaries in Scope of...		4	26	2022-08-07, 12:49...	EAR	2022-09-26, 9:25...
Financial Barriers		2	2	2022-08-07, 12:44...	EAR	2022-08-07, 10:07..
Knowledge of Patient F...		8	24	2022-08-07, 12:49...	EAR	2022-08-07, 12:49..
Time Constraints		5	15	2022-08-07, 12:49...	EAR	2022-08-07, 10:03..
2. COVID-19		6	19	2022-08-07, 12:40...	EAR	2022-08-07, 12:41..
Impact on Patient Care		9	21	2022-08-07, 2:16 PM	EAR	2022-08-07, 2:17 PI
Impact on Quality of Tr...		3	4	2022-08-07, 2:17 PM	EAR	2022-08-07, 2:17 PI
Virtual Care		2	4	2022-08-07, 2:16 PM	EAR	2022-08-07, 2:16 PI
3. Curriculum Development		8	51	2022-08-07, 12:41...	EAR	2022-08-07, 12:41..
Addictions Training		9	77	2022-08-07, 2:25...	EAR	2022-08-07, 3:22...
Centered in Family Med...		7	20	2022-08-07, 2:26...	EAR	2022-08-07, 10:03..
Counselling-Psychothe...		9	94	2022-08-07, 2:26...	EAR	2022-08-07, 10:34..
Implementation Methods		3	10	2022-08-07, 2:39...	EAR	2022-08-07, 2:40...
Learning Improvement		9	83	2022-08-07, 2:41 PM	EAR	2022-08-07, 2:41 PI
Longitudinal		4	9	2022-08-07, 2:40...	EAR	2022-08-07, 2:40...
Positive Learning Ex...		4	12	2022-08-07, 2:43...	EAR	2022-08-07, 2:43...
Transitionary phases		3	7	2022-08-07, 2:42...	EAR	2022-08-07, 2:43...
Limited duration of resi...		4	7	2022-08-07, 2:28...	EAR	2022-08-07, 2:29...
4. Professinoal Identity &...		4	23	2022-08-07, 12:41...	EAR	2022-08-07, 12:41..
Expert-Expertise		4	39	2022-08-07, 2:43...	EAR	2022-08-09, 9:32...
Common Presentation		7	28	2022-08-07, 10:08...	EAR	2022-08-07, 10:09..

	Modified By	Color
..	EAR	
..	EAR	
.	EAR	
.	EAR	
..	EAR	
..	EAR	
..	EAR	
.	EAR	
M	EAR	
M	EAR	
M	EAR	
.	EAR	
	EAR	
..	EAR	
..	EAR	
	EAR	
M	EAR	
	EAR	
	EAR	
	EAR	
	EAR	
.	EAR	
.	EAR	
..	EAR	

Personal Interest	3	7	2022-08-09, 9:32...	EAR	2022-06-15, 8:43...
Family Physician role-q...	6	22	2022-08-07, 2:45...	EAR	2022-08-07, 2:46...
Relationship betwee...	3	4	2022-08-07, 2:46...	EAR	2022-08-07, 2:46...
Stigma and acces...	3	3	2022-08-07, 2:46...	EAR	2022-08-07, 2:47...
Formation of Professio...	5	19	2022-08-07, 2:44...	EAR	2022-08-07, 2:45...
Ranges of clinical com...	6	33	2022-08-07, 2:43...	EAR	2022-08-07, 2:44...

EAR
EAR
EAR
EAR
EAR
EAR
